# Enhanced Recovery After Surgery can Improve Patient Outcomes and Reduce Hospital Cost of Gastrectomy for Cancer in the West: A Propensity-Score-Based Analysis

**DOI:** 10.1245/s10434-021-10079-x

**Published:** 2021-05-14

**Authors:** Jacopo Weindelmayer, Valentina Mengardo, Angela Gasparini, Michele Sacco, Lorena Torroni, Mauro Carlini, Giuseppe Verlato, Giovanni de Manzoni

**Affiliations:** 1 General and Upper G.I. Surgery Division, University of Verona, Borgo Trento, Verona, Italy; 2 Department of Diagnostics and Public Health, University of Verona, Verona, Italy; 3 Anesthesia and Intensive Care Unit, Department of Surgery, Dentistry, Pediatrics and Gynecology, University of Verona, Verona, Italy

## Abstract

**Background:**

Data on ERAS for gastrectomy are scarce, and the majority of the studies come from Eastern countries. Patients in the West are older and suffer from more advanced tumors that impair their clinical condition and often require neoadjuvant treatment. This retrospective study assessed the feasibility and safety of an Enhanced Recovery After Surgery (ERAS) protocol for gastrectomy in a Western center.

**Methods:**

We conducted a single-center study of 351 patients operated for gastric cancer: 103, operated from January 2015 to December 2016, followed the standard pathway, while 248, operated from January 2017 to December 2019, followed the ERAS program. The primary outcomes considered were length of hospital stay (LOS) and direct costs. Secondary outcomes were 90-day morbidity and mortality, readmission rate, and compliance with ERAS items. A propensity score (PS) was built on confounding variables.

**Results:**

Compliance with ERAS items after the program was ≥ 70%. Univariable analysis evidenced a 2-day median reduction in LOS and a median cost reduction of €826 per patient in the ERAS group. PS-based multivariable analysis confirmed a significant, 2-day decrease in median LOS and a €1097 saving after ERAS introduction. Ninety-day mortality decreased slightly in ERAS group, while complications and readmissions did not change significantly. When complications were included in the multivariable analysis, ERAS retained its significance, although the effects on LOS and cost were blunted to a median reduction of 1 day and €775, respectively.

**Conclusions:**

ERAS for gastrectomy improved patients’ recovery and reduced hospital costs without changes in morbidity, mortality, or readmission.

**Supplementary Information:**

The online version contains supplementary material available at 10.1245/s10434-021-10079-x.

Evidence about the effectiveness of Enhanced Recovery After Surgery (ERAS) for gastrectomy is still scarce and mostly comes from Eastern experiences. Although guidelines for the enhanced recovery pathway after gastrectomy were published in 2014 by the ERAS Society,^1^ its application worldwide was probably limited by the complexity of this operation. Moreover, despite increasing evidence that high-volume centers for gastrectomy have better postoperative outcomes,^2^ many Western countries have no centralization policy for gastric cancer, thus leading to a large number of centers performing few operations. This could have led to less standardization in the perioperative pathways and the implementation of formal ERAS programs for gastrectomy. Papers from Eastern countries report encouraging results with ERAS, evidencing a reduction in hospitalization, morbidity, and mortality.^3^–^6^ Nevertheless, it is not straightforward to translate these results into everyday Western practice, because European and US patients are generally older, exhibit multiple chronic conditions, and have tumors at a more advanced stage that often require neoadjuvant treatment and more extended surgery.^7^–^10^ Therefore, it is of primary importance to obtain information from Western centers on the feasibility and safety of ERAS for gastrectomy.

We designed a retrospective cohort study of a large group of gastric cancer patients who underwent gastrectomy before and after introduction of an ERAS protocol for gastrectomy. To counterbalance differences between the two periods, a propensity score (PS) was designed on confounding variables and used to investigate the PS-adjusted effect of ERAS on the selected outcomes.

## Patients and Methods

Three hundred ninety-five patients underwent (total or subtotal) gastrectomy for gastric adenocarcinoma at our institution from January 2015 to December 2019. Seventeen patients who underwent palliative (e.g., palliative gastrojejunal bypass, feeding jejunostomy, and explorative laparotomy) and/or emergency surgery or were treated with concomitant hyperthermic intraperitoneal chemotherapy were excluded as unsuitable for the standard postoperative pathway. Twenty-seven additional patients with R2 resection were not included in the study, thus leaving 351 patients for analysis. During the study period, three experienced surgeons performed all the operations in accordance with the latest international guidelines available for gastric cancer.^11^–^13^ In particular, D1+ lymphadenectomy was performed for early gastric cancer, while D2 or extended D2 lymphadenectomy was carried out in advanced tumors. Seventeen patients with oligometastatic gastric cancer who underwent radical gastrectomy after intensive chemotherapy were included. As ERAS for gastrectomy was fully adopted at our institution in January 2017, patients treated before this date were included in the standard group, while patients operated thereafter were assigned to the ERAS group. Standard and ERAS protocols are fully described in the Electronic Supplementary Material, and Table 1 summarizes the differences between the two pathways. Data were prospectively collected and retrospectively analyzed.

**Table 1:** Standard and ERAS items considered

	Standard protocol	Enhanced recovery after surgery protocol
*Preoperative*		
Counseling	Pulmonary prehabilitation	Pathway explanation and informative booklet. Nutritional counseling and physiotherapy prehab
Preoperative fasting	Ten hours for solids and 8 h for clear fluids before surgery	Carbohydrate load (preop, Nutricia) 12 and 2 h before surgery
*Intraoperative*		
Analgesia	Not standardized	Multimodal: TEA for open surgery or RSB and/or subcostal TAP block for laparoscopic surgery + CNS-targeted drugs
Prophylaxis	Antibiotic prophylaxis, VTE (pharmacological and mechanical)	Antibiotic prophylaxis, VTE (pharmacological and mechanical), PONV prophylaxis
Fluids	Not standardized	Goal-directed fluid management
Extubation	Immediate extubation	Immediate extubation
Hospital acuity	Ward; PCU for close monitoring/respiratory need	Ward; PCU for close monitoring/respiratory need
NGT	Remove on POD 1	Remove at end of surgery
*Postoperative*		
Analgesia	Not standardized	Multimodal: TEA; fixed time interval-opioid sparing analgesia + rescue therapy with NSAIDs or codeine
Fluid	Not standardized	Zero balance goal; stop iv fluids within POD 4
Abdominal drain	Always placed. No routine anastomotic leak test. Remove on POD 3–4	Placed only after TG. No routine anastomotic leak test. Remove on POD 3
Line management	Not standardized	Remove urinary catheter on POD 2. Remove peridural catheter on POD 4
Diet	Not standardized	POD 1 clear fluids; POD 2–5 nutritional counselling; POD 3 soft diet
Rehabilitation	Not standardized	POD 1–3 pulmonary physiotherapy; POD 1 chair and bedside exercise; POD 2–3 assisted ambulation
Length of stay	Not standardized	POD 6 if discharge criteria are met (timed discharge)

*CNS* central nervous system, *TEA* thoracic epidural anesthesia, *PONV* postoperative nausea and vomiting, *RSB* rectus sheath block, *TAP* transversus abdominis plane, *VTE* venous thromboembolism, *PCU* progressive care unit, *NGT* nasogastric/jejunal tube, *POD* postoperative day, *TG* total gastrectomy

### Outcomes

The primary outcomes were length of hospital stay (LOS) and direct cost, while the secondary outcomes considered were 90-day morbidity and mortality, 90-day readmission rate, the need for postdischarge care, and enteral support at home. Compliance with ERAS items was used as a quality control for protocol application; the threshold was set at 70%.

Direct hospital costs were calculated in euros (€) by the healthcare administrative clinical department, starting from the preoperative assessment until 30 days after discharge, including, if present, readmission and reoperation costs within 90 days from the operation. Costs comprised preoperative functional assessment, operating-room costs, housing costs, medical and nursing care, medication, laboratory assessments, and imaging.

Morbidity was considered as any complication that occurred within 90 days after the operation. Complications were graded according to the Clavien-Dindo (CD) classification^14^ and divided into mild (CD 1–2) and severe (CD 3–5).

Compliance with the protocol was evaluated by 12 ERAS items, 6 collected in the pre-intraoperative period and 6 in the postoperative period. Pre-intraoperative items included carbohydrate load, multimodal analgesia, goal-directed fluid therapy, nasogastric tube/jejunal (NGT) avoidance, immediate extubation, and transfer to a surgical ward. Postoperative items comprised postoperative day (POD) 2 urinary catheter removal, early resumption of liquid and soft diet, targeted physiotherapy (on chair and ambulation), and no drain placement in subtotal gastrectomy or early drain removal in total gastrectomy.

### Statistical Analysis

The bulk of the LOS distribution was confined to a few discrete values with a minimum of 5 days and the 75th percentile at 8–9 days with several outliers. Hence, LOS was dichotomized into on-time (5–6 days) and late (> 6 days) for multivariable and PS-adjusted statistical analyses.

Relevant variables were compared between the standard and ERAS groups using the Fisher exact test or chi-square test as appropriate for nominal variables, and by the Wilcoxon–Mann–Whitney test for continuous variables with skewed distribution.

PS was estimated using a logit model on the following confounding variables: sex, age, body mass index (BMI), smoking habits, previous major surgery, American Society of Anesthesiologists (ASA) class, clinical stage, neoadjuvant treatment, type of gastrectomy, use of minimally invasive surgery, and type of lymphadenectomy (Supplementary Fig. S1). PS was then used as a covariate in multivariable analyses.

The effect of ERAS implementation was evaluated by quantile regression for quantitative outcomes (LOS and cost), and by logistic regression for dichotomous outcomes (timed discharge, readmission rate, complications, and mortality).

Considering the increased use of laparoscopic surgery in the ERAS group (11% versus 1%, *p* = 0.001), minimally invasive gastrectomy was considered a possible cause of significant uncertainty. Therefore, to test the robustness of the results, sensitivity analysis was conducted excluding patients treated with a minimally invasive procedure. A modified propensity score (mPS) was estimated using a logit model that included all PS confounding variables with the exclusion of minimally invasive surgery, and multivariable analysis was conducted to evaluate the effect of ERAS implementation.

Missing data were very few and affected only secondary analyses, so no deletion or imputation methods were necessary. Significance was set at 0.05. Statistical analysis was conducted using Stata software version 16.0 (StataCorp, College Station, TX); in particular, PS was estimated by using the pscore command.

## Results

### Patient and Treatment Features

Alcohol abuse and diabetes were more prevalent in the standard group, while previous major surgery was more common in the ERAS group (Table 2). High-risk surgical patients, defined as ASA III–IV, were evenly distributed between the two groups (34% versus 32%). An increasing number of proximal tumors and advanced clinical-stage tumors (cStage 3–4) were found in the ERAS group. About 50% of the whole cohort underwent D2 lymphadenectomy, with greater use of extended (D2+ or higher) lymphadenectomy in ERAS patients (35%) compared with standard patients (22%).

**Table 2:** Patient and treatment features of the study groups

	Standard group (*n* = 103)	ERAS group (*n* = 248)	*p* value
Sex, female (%)	48 (47)	94 (38)	0.131
Age, median (p25–p75) (years)	70 (60–78)	68 (60–76)	0.437
BMI, median (p25–p75) (kg/m^2^)	25 (22–28)	25 (22–28)	0.787
*Smoking history* (%)			0.259
No	60 (58)	149 (60)	
Active	19 (19)	30 (12)	
Former	24 (23)	69 (28)	
Alcohol abuse (%)	**6 (6)**	**3 (1)**	**0.021**
Preoperative albumin, median (p25–p75)	36 (33–39)	36 (33–39)	0.195
*Comorbidities (%)*			
Cardiovascular	60 (58)	148 (60)	0.805
Respiratory	5 (5)	26 (10)	0.101
Diabetes	**36 (35)**	**60 (24)**	**0.040**
Kidney	9 (9)	16 (6)	0.495
Previous major surgery	**9 (9)**	**48 (19)**	**0.016**
ASA III–IV	35 (34)	79 (32)	0.699
*Oncological features (%)*			
Histology, adenocarcinoma	103 (100)	248 (96)	0.068
*Location*			**0.039**
Proximal	**12 (12)**	**59 (24)**	
Body	**39 (38)**	**90 (36)**	
Antrum	**49 (47)**	**95 (38)**	
Remnant	**3 (3)**	**4 (2)**	
*Clinical stage*			**0.001**
0–I	**34 (33)**	**58 (23)**	
II	**29 (28)**	**50 (20)**	
III	**40 (39)**	**123 (50)**	
IV	**0 (0)**	**17 (7)**	
Neoadjuvant therapy	**19 (18)**	**100 (40)**	**< 0.001**
*Surgical features *(%)			
*Type of surgery* (%)			0.366
STG	50 (48)	104 (42)	
TG	48 (47)	123 (50)	
TG + DE	5 (5)	21 (8)	
*Lymphadenectomy* (%)			**0.003**
D1+	**29 (28)**	**35 (14)**	
D2	**51 (50)**	**126 (51)**	
D2+	**23 (22)**	**87 (35)**	
Nodal harvesting, median (p25–p75)	**37 (28–47)**	**43 (33.5–55)**	**< 0.001**
Extended organ resection (%)	17 (16)	33 (13)	0.435
Minimally invasive surgery (%)	**1 (1)**	**27 (11)**	**0.001**

Significant results highlighted in bold

*STG* subtotal gastrectomy, *TG* total gastrectomy, *TG + DE* total gastrectomy + distal esophagectomy

Regarding treatment, greater use of neoadjuvant therapy and minimally invasive surgery was observed in the ERAS group with respect to the standard group. However, while neoadjuvant treatment was administered to 40% of ERAS patients, laparoscopic gastrectomy was still seldom used.

### ERAS Item Adherence

**Fig. 1:**
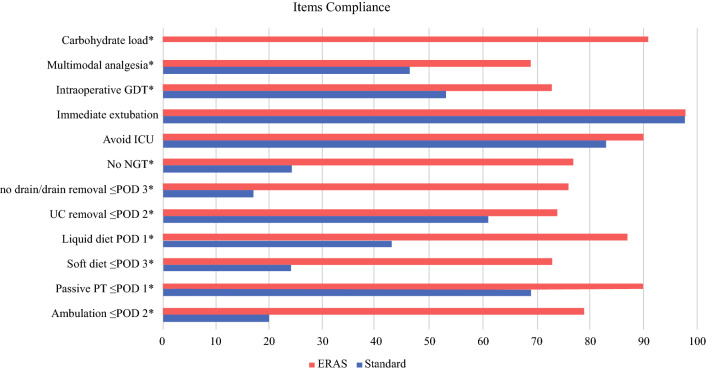
Comparison between ERAS and standard group on compliance with perioperative and postoperative items (success rate)*. GDT* goal directed therapy, *ICU* intensive care unit, *NGT* nasogastric/jejunal tube, *UC* urinary catheter, *PT* physiotherapy, *POD* postoperative day. *Significant results

Adherence to ERAS after its implementation was appropriate for all the items, reaching achievement of 70% or higher (Fig. 1). While immediate extubation and direct ward transfer were already realized in most of the standard group patients, analgesia optimization and use of intraoperative goal-directed fluid therapy significantly increased after ERAS introduction. All postoperative items improved significantly in the ERAS group, with adherence ranging from 73% for resumption of soft diet to 90% for early mobilization and resumption of physiotherapy.

### Univariable Analysis

Univariable analysis (Table 3) evidenced a 2-day median reduction in LOS after ERAS introduction. The total cost decreased accordingly from a median of €7800 to €7000 per patient, as a result of a reduction in ward, laboratory test, and radiology costs. Interestingly, not only did the median total cost decrease, but so did the interquartile range, from €3683 to €2171. No difference between the two groups was found in terms of complication rate, readmission rate, or facilities or enteral support at home. It should be remembered, however, that facilities and support at home were rarely prescribed. Ninety-day mortality was rare in both groups, accounting for less than 2% of the total (seven patients). Nonetheless, univariable analysis suggests a possible reduction in mortality after ERAS implementation (0.8% versus 4.8%, *p* = 0.025).

**Table 3:** Postoperative outcomes in the Standard and ERAS groups

	Standard group (*n* = 103)	ERAS group (*n* = 248)	*p* value
Length of stay, median (p25–p75) (days)	**8 (7–9)**	**6 (6–8)**	**< 0.001**
Timed discharge (%)	**16 (15)**	**128 (52)**	**< 0.001**
*Complications, mild/severe* (%)			
Anastomotic leak	0/0 (0/0)	1/2 (0.4/0.8)	1
Pulmonary	3/5 (2.9/4.8)	8/13 (3.2/5.2)	1
Cardiac	9/2 (8.7/1.9)	15/0 (6/0)	0.062
Other	38/10 (36.9/9.7)	85/23 (34.3/9.3)	0.870
Total	35/16 (34/15.5)	84/34 (33.9/13.7)	0.895
90-Day mortality (%)	**5 (4.8)**	**2 (0.8)**	**0.025**
90-Day readmission (%)	9 (8.8)	16 (6.4)	0.494
Postdischarge care (%)	9 (8.8)	10 (4.2)	0.078
Enteral support at home (%)	7 (6.9)	6 (2.4)	0.061
*Direct costs €, median* (*p*25–*p*75)			
Surgery	2977 (2545–3391)	2987 (2665–3342)	0.719
Ward	**3500 (3100–5900)**	**3450 (3100–4350)**	**0.022**
ICU	0 (0–0)	0 (0–0)	0.097
Laboratory tests	**262 (191–391)**	**233 (180–233)**	**0.049**
Radiology	**75 (25–263)**	**50 (25–103)**	**0.016**
Other	88 (70–131)	88 (70–150)	0.235
Total	**7852 (6327–10,010)**	**7026 (6259–8430)**	**0.012**

Mild complication: Clavien–Dindo class I or II; severe complication: Clavien–Dindo class III or above

Significant results highlighted in bold

### Multivariable Analysis

Multivariable analysis was first conducted considering only ERAS application and PS (Table 4). ERAS was associated with a significant, 2-day decrease in median LOS, a sixfold increase in the probability of timed discharge, and a €1097 reduction in total cost. Ninety-day mortality decreased after ERAS introduction, while complications and readmission did not change significantly. However, note that the number of postoperative deaths was very small (*n* = 7), making estimates imprecise. Of note, PS was not significantly associated with any of the six outcomes considered.

**Table 4:** Effect of ERAS application and propensity score on LOS, total costs, timed discharge, complications, readmission rate, and mortality

	ERAS versus standard group		Per one unit increase in PS	
Quantile regression	Coefficient (95% CI)	*p* value	Coefficient (95% CI)	*p* value
Length of stay, days	**–2 (–2.59 to –1.41)**	**< 0.001**	0 (–1.48 to 1.48)	1
Total cost, euros	**–1097 (–1732 to –462)**	**0.001**	1013 (–579 to 2605)	0.211

Logistic regression	Odds ratio (95% CI)	p value	Odds ratio (95% CI)	*p* value
Timed discharge	**6.22 (3.30–11.71)**	**< 0.001**	0.66 (0.17–2.59)	0.550
Complications	0.86 (0.52–1.42)	0.544	1.62 (0.46–5.75)	0.454
Readmission	0.67 (0.26–1.72)	0.406	1.45 (0.12–17.23)	0.769
90-Day mortality	**0.09 (0.01–0.54)**	**0.009**	41.97 (0.32–5492.98)	0.133

Statistical analysis performed by quantile regression model for quantitative outcomes and by logistic regression model for binary outcomes

Significant results highlighted in bold

*PS* propensity score

When complications were included in multivariable analysis for LOS and total cost (Table 5), ERAS retained its significance with a median reduction of 1 day and €775 compared with standard treatment. Notably, complications, when present, completely nullified the gains in patients’ management obtained with ERAS: indeed, the increase in LOS and cost associated with severe complications were 7–10 times greater than the decrease yielded by ERAS.

**Table 5:** Multivariable analysis on LOS and total cost considering group, complications (mild and severe), and PS. Statistical analysis performed using quantile regression model

	Length of stay		Total cost	
	Coefficient (95% CI)	*p* value	Coefficient (95% CI)	*p* value
*Group*				
Standard	1		1	
ERAS	**–1 (–1.67 to –0.33)**	**0.003**	**–775 (–1360 to –190)**	**0.010**
*Complications*				
No	1		1	
Mild	**1 (0.39–1.61)**	**0.001**	**1617 (1080–2155)**	**< 0.001**
Severe	**7 (6.16–7.84)**	**< 0.001**	**6235 (5506–6963)**	**< 0.001**
Propensity score	0 (–1.68 to 1.68)	1	1131 (–337 to 2598)	0.131

Significant results highlighted in bold

### Sensitivity Analysis

Sensitivity multivariable analysis excluding minimally invasive surgery patients (28 patients) confirmed the results of the primary analysis. When considering only ERAS and mPS, a slight reduction in the effect of ERAS on LOS (1.52 versus 2 days decrease) was noted (Supplementary Table S1). Nevertheless, when complications were included in the analysis for LOS and total cost, the effect of ERAS and complications remained roughly the same (Supplementary Table S2).

## Discussion

This study represents the largest single-center review of ERAS for gastrectomy in the West to date. It confirmed the feasibility of ERAS for gastrectomy in Western patients and evidenced that its application can lead to a significant reduction in length of hospital stay and direct costs. Moreover, it confirms the safety of ERAS as neither complications nor readmission rate increased after its introduction and mortality was rare in both groups.

Our findings are consistent with two recently published metaanalyses conducted by Changsheng (15 RCTs)^15^ and Wee (18 RCTs, 8 observational studies)^16^ that evidenced shorter LOS and reduced hospitalization costs with similar postoperative morbidity and mortality after ERAS implementation. On the other hand, they observed an increased risk of readmission in the ERAS group.

Despite the strength of evidence provided by these metaanalyses, it should be borne in mind that all the considered studies came from Eastern countries, except for one prospective cohort series on 252 patients from the UK^17^ that included not only gastrectomies but also esophagectomies. It is well established that gastric cancer in Eastern countries is often detected at an early stage during screening tests, and therefore the treatment of choice in the majority of patients is laparoscopic primary surgery. Moreover, patients are usually younger and in good clinical condition, thus possibly reducing the postoperative risk of complications. Therefore, results from these studies are not sufficient to draw definitive conclusions on ERAS for gastrectomy in the West.

A 2018 retrospective study from the USA compared an ERAS group with a historical control using a propensity score in a small cohort of patients (*n* = 96).^18^ This reported encouraging results with a significant reduction in LOS and similar incidence of complications. Nevertheless, this study, like the majority of studies on ERAS for gastric cancer surgery, did not report any data on compliance with the protocol. The importance of compliance is often underrated in ERAS studies, but it represents a quality control for implementation of the protocol and its importance increases with the complexity of the operation as it becomes more difficult to complete the postoperative pathway. A prospective multicenter observational study, conducted in seven Italian centers, that aimed to evaluate compliance with ERAS items after gastrectomy evidenced high variability in ERAS application with several items reaching a very low adherence rate.^7^

In our study, compliance of at least 70% for each ERAS item was observed, which was considered appropriate due to the variability caused by nonmodifiable factors such as patient characteristics, hospital organization limits, and complications.

The advantage of ERAS in terms of direct hospital costs has been described previously in some series^16^–^20^ and was confirmed in our study, with a median saving per patient of approximately €1000. Nevertheless, complications remained the main determinant of cost increases, leading to a 7–10-fold increment when a severe complication occurs, a result consistent with a recent study on esophagectomy conducted in our department.^19^

Lastly, although the majority of patients underwent open gastrectomy due to their advanced clinical stage or surgeon preference, ERAS items were applied with good compliance, obtaining satisfying results. It is possible that the effect of ERAS may be more evident in open surgery, as suggested by Huang,^21^ but it is also likely that obtaining good compliance in these patients will be more challenging, especially for postoperative items.

This study has some limitations. Temporal bias between the two groups could have altered the effect of ERAS on the selected outcomes, although the use of a PS in the analysis helped to reduce the difference between the two cohorts. Moreover, the limited number of events does not allow reliable conclusions to be drawn on mortality, even if a slight advantage in the ERAS group was noted.

## Conclusions

This study demonstrates the feasibility and safety of an ERAS protocol for gastrectomy in a large Western series. The use of ERAS can improve patients’ recovery and reduce hospital costs without increasing readmission or the need for postdischarge care. Based on this study and evidence from literature, implementation of ERAS for gastrectomy in Western centers should be supported.

## Supplementary Information

Below is the link to the electronic supplementary material.

Supplementary file1 (DOCX 47 kb)
