# Supplementary material for: Enhanced Recovery After Surgery can Improve Patient Outcomes and Reduce Hospital Cost of Gastrectomy for Cancer in the West: A Propensity-Score-Based Analysis
Source: Ann Surg Oncol. 2021 May 14;28(12):7087–94. doi: 10.1245/s10434-021-10079-x (PMC8519899; doi:10.1245/s10434-021-10079-x)
Supplement: Supplementary file 1 — Supplementary file1 (DOCX 47 kb) [file 10434_2021_10079_MOESM1_ESM.docx]

***Supplementary material***

**Detailed description of Standard and ERAS protocols**

*Standard protocol*

Standard preoperative protocol included an outpatient preoperative evaluation with the surgeon and a respiratory prehabilitation. Preoperative fasting started 10 hours before surgery for solids and 8 hours for clear fluids. Peridural catheter placement and intraoperative fluid management were managed as per anesthetist preference. At the end of the operation a prophylactic drain was routinely placed in both total ad subtotal gastrectomy. Nasogastric/nasojejunal tube (NGT) was routinely used for decompression up to 24 hours after the operation. Immediate extubation and transfer to the surgical ward or to a progressive care unit (PCU), equipped with close monitoring and noninvasive ventilation support, without intervening of ICU care was carried out whenever possible. Patient was evaluated by a physiotherapist on POD 1 and started the rehabilitation program on the same day, beginning with lung exercise at bed and chair and progressing to active assisted movement when tolerated. The prophylactic drain was removed on POD 3-4 if no suspicious debt was noted. Liquid diet was started on POD 2-3 and soft diet with supplement drinks was started on POD 4-5 if tolerated. Nutrition counselling was considered only in selected cases. No standardized goal for discharge was set.

*ERAS protocol*

Since January 2017 ERAS for gastric cancer has been adopted for all patients regardless of stage and previous oncological treatment. However, it was not utilized with multiorgan resection, HIPEC and emergency operation. Patient was informed about the pathway during the outpatient surgical assessment with the use of an informative booklet. Nutritional counselling and physiotherapy prehabilitation (walking and pulmonary exercises) were carried out during preoperative workup. Carbohydrate load (Nutricia-preop) was administered, if tolerated, 12 and 2 hours before the operation to avoid prolonged fasting. Multimodal anesthesia and analgesia were applied by a dedicated anesthesiology team through the following principles:

- PONV prophylaxis;
- Intraoperative goal directed fluid management (described in a previous

publication for esophagectomy);

- Reversal of neuromuscular blockade
- Thoracic epidural analgesia for open surgery and rectus sheath block or subcostal transversus abdominis plane block for laparoscopic surgery;
- Use of short active medications associated with additional drugs with specific

central nervous system targets (magnesium sulphate, dexmedetomidine or ketamine) to minimize opioid use.

Placement of abdominal drain was planned only for total gastrectomy while no drain was routinely placed in subtotal gastrectomy. NGT was not routinely placed unless intraoperative complications occurred. Consistently with the Standard protocol, patient was immediately extubated and transferred to the surgical ward or PCU whenever possible. On POD 1 patient could drink clear fluids and sip a supplemental nutrition drink if tolerated. Nutrition counseling was scheduled for all the patients on POD 2 and a personalized, fractionated diet with increasing consistence (starting from strained and pureed up to solid) was started on POD 3. Postoperative physiotherapy was started on POD 1 with chair mobilization and bedside exercise and increased on POD 2 with assisted ambulation whenever tolerated by the patient. Discharge was planned for POD 6 based on the following criteria: autonomy in mobilization, pain controlled by oral analgesics, tolerability of oral nutrition and/or enteral nutrition of at least 60% of the daily target requirement [7]. Systematic audit within the ERAS team was carried out on a monthly basis.

**Figure S1.** Propensity score of Standard (Untreated) and ERAS (Treated) stratified in 5 balanced blocks and based on the following variables: sex, age, BMI, smoking habits, previous major surgery, ASA class, clinical stage, neoadjuvant treatment, type of gastrectomy, use of minimally invasive surgery and type of lymphadenectomy. Mean PS 0.71, SD 0.18.

**Table S1**. Effect of ERAS application and modified propensity score on LOS, total costs, timed discharge, complications, readmission rate and mortality. Statistical analysis was performed by quantile regression model for quantitative outcomes, and by logistic regression model for binary outcomes.

|  | ERAS vs standard group | |  | Per one unit increase in mPS | |
| --- | --- | --- | --- | --- | --- |
| **Quantile regression** | Coefficients (95% CI) | p value |  | Coefficients (95% CI) | p value |
| Length of stay, days | **-1.52 (-2.11 - -0.93)** | **<0.001** |  | 1.48 (-0.12 – 3.08) | 0.069 |
| Total cost, euros | **-1040 (-1701 - -379)** | **0.002** |  | 645 (-1142 – 2432) | 0.478 |
|  |  |  |  |  |  |
| **Logistic regression** | Odds ratio (95% CI) | p value |  | Odds ratio (95% CI) | p value |
| Timed discharge | **6.18 (3.25-11.74)** | **<0.001** |  | 0.37 (0.08-1.68) | 0.198 |
| Complications | 0.88 (0.53-1.46) | 0.625 |  | 1.24 (0.31-4.88) | 0.760 |
| Readmission | 0.66 (0.26-1.68) | 0.386 |  | 2.39 (0.17-34.45) | 0.522 |
| 90-days mortality | **0.10 (0.02-0.59)** | **0.011** |  | 74.44 (0.43-12745.65) | 0.100 |

mPS: modified propensity score (without minimally invasive surgery variable)

Significant results are highlighted in bold.

**Table S2**: Multivariable analysis on LOS and total cost considering group, complications (Mild and Severe) and modified propensity score. Statistical analysis was performed using quantile regression model.

|  | **Length of stay** | | | |  | | **Total cost** | | |
| --- | --- | --- | --- | --- | --- | --- | --- | --- | --- |
|  | Coefficients (95% CI) | | p value | |  | | Coefficients (95% CI) | | p value |
| Group | |  | |  | |  | |  |  |
| … Standard | | 1 | |  | |  | | 1 |  |
| … ERAS | | **-1 (-1.69 - -0.31)** | | **0.005** | |  | | **-683 (-1347 - -19)** | **0.044** |
| Complications | |  | |  | |  | |  |  |
| … No | | 1 | |  | |  | | 1 |  |
| … Mild | | **2 (1.33 – 2.67)** | | **0.001** | |  | | **1466 (825 – 2108)** | **<0.001** |
| … Severe | | **7 (6.13 – 7.87)** | | **<0.001** | |  | | **6291 (5463 – 7119)** | **<0.001** |
| mPS | | 0 (-1.88 – 1.88) | | 1 | |  | | 704 (-1095 – 2504) | 0.442 |

mPS: modified propensity score (without minimally invasive surgery variable)

Significant results are highlighted in bold.
